# Risk of nosocomial coronavirus disease 2019: comparison between single- and multiple-occupancy rooms

**DOI:** 10.1186/s13756-024-01454-w

**Published:** 2024-08-30

**Authors:** Hyeon Jae Jo, Pyoeng Gyun Choe, Ji Seon Kim, Mimi Lee, Minkyeong Lee, Jiyeon Bae, Chan Mi Lee, Chang Kyung Kang, Wan Beom Park, Nam Joong Kim

**Affiliations:** 1 Department of Internal Medicine, Seoul National University College of Medicine, 101 Daehak-ro Jongno-gu, Seoul, 03080 Republic of Korea; 2 Infection Control Office, Seoul National University Hospital, Seoul, Republic of Korea

**Keywords:** COVID-19, Nosocomial, Transmission, Respiratory virus, Hospitals

## Abstract

**Background:**

There is an ongoing controversy regarding whether single-occupancy rooms are superior to multiple-occupancy rooms in terms of infection prevention. We investigated whether treatment in a multiple-occupancy room is associated with an increased incidence of nosocomial coronavirus disease 2019 (COVID-19) compared with treatment in a single-occupancy room.

**Methods:**

In this retrospective cohort study, every hospitalization period of adult patients aged ≥ 18 years at a tertiary hospital in Korea from January 1, 2022, to December 31, 2022, was analyzed. If COVID-19 was diagnosed more than 5 days after hospitalization, the case was classified as nosocomial. We estimated the association between the number of patients per room and the risk of nosocomial COVID-19 using a Cox proportional hazards regression model.

**Results:**

In total, 25,143 hospitalizations per room type were analyzed. The incidence rate of nosocomial COVID-19 increased according to the number of patients per room; it ranged from 3.05 to 38.64 cases per 10,000 patient-days between single- and 6-bed rooms, respectively. Additionally, the hazard ratios of nosocomial COVID-19 showed an increasing trend according to the number of patients per room, ranging from 0.14 (95% confidence interval 0.001–1.03) to 2.66 (95% confidence interval 1.60–4.85) between single- and 6-bed rooms, respectively.

**Conclusions:**

We demonstrated that the incidence of nosocomial COVID-19 increased according to the number of patients per room. To reduce nosocomial infections by respiratory viruses, the use of multiple-occupancy rooms should be minimized.

**Supplementary Information:**

The online version contains supplementary material available at 10.1186/s13756-024-01454-w.

## Introduction

Nosocomial spread of severe acute respiratory syndrome coronavirus 2 (SARS-CoV-2) was reported during the coronavirus disease 2019 (COVID-19) pandemic [1, 2]. To prevent nosocomial spread, many hospitals implemented additional strategies beyond the standard precautions. These included testing all patients on admission, improving ventilation, ensuring universal masking, encouraging vaccination of patients and healthcare workers, and isolating patients with confirmed COVID-19 [3, 4].

Patients admitted to multiple-occupancy rooms have a higher risk of encountering other patients with transmissible infectious diseases relative to patients in single-occupancy rooms [5, 6]. Some studies have demonstrated that the use of single-occupancy rooms significantly reduces the rates of colonization of multidrug-resistant organisms (MDROs) and healthcare-associated infection, such as bloodstream infection or *Clostridium* *difficile* infection, compared with treatment in multiple-occupancy rooms [7–9]. However, there is still controversy regarding the advantages of single-occupancy rooms in reducing multidrug-resistant organism colonization and healthcare-associated infection. This controversy has arisen because most previous studies had low levels of evidence and included many confounding variables, thus hindering interpretation [10–13].

MDROs mainly spread via contaminated hands and the environment. In contrast, respiratory viruses, including influenza virus and SARS-CoV-2, mainly spread by droplets or aerosols. Few studies have examined the impact of multiple-occupancy rooms on nosocomial transmission of respiratory viruses. In one previous study, the incidences of nosocomial influenza were 2.0 and 0.7 for 100 patient-days in double- and single-occupancy rooms, respectively [5]. Several studies have revealed that treatment in multiple-occupancy rooms is a risk factor for nosocomial COVID-19 [14–18]. This study aimed to investigate the impact of multiple-occupancy rooms on the incidence of nosocomial COVID-19.

## Methods

### Study setting

This retrospective observational study was conducted at a tertiary hospital in Seoul, South Korea. This is an 1803-bed university-affiliated hospital with 1367 non-intensive care unit beds for adults, 126 (9.2%) single-bed rooms, 364 (26.6%) 2-bed rooms, 39 (2.9%) 3-bed rooms, 184 (13.5%) 4-bed rooms, 120 (8.8%) 5-bed rooms, and 534 (39.0%) 6-bed rooms. In multiple-occupancy rooms, the beds were placed 7 feet apart and separated by curtains. Among the 126 single-bed rooms, 35 (27.8%) were located in wards with only single-bed rooms, while 91 (72.2%) were located in wards with both single- and multi-bed rooms. This study was performed from January 1, 2022, to December 31, 2022, when the number of confirmed COVID-19 cases was at its peak in Korea. The Delta variant was dominant until January 2022; thereafter, the Omicron BA.1, BA.2, and BA.5 variants were dominant [19].

During the study period, a SARS-CoV-2 polymerase chain reaction (PCR) assay was performed before hospitalization of all patients, and patients were admitted after a negative result had been confirmed. If the SARS-CoV-2 PCR assay result was positive on admission for patients whose admission was inevitable, those patients were isolated in single-occupancy rooms. Visitors’ access was restricted to individuals with a negative PCR test result obtained within 48 h. Universal masking of patients and healthcare workers was implemented, and vaccination of patients and healthcare workers was encouraged. In addition to screening for all admissions, the SARS-CoV-2 PCR assay was repeated if patients had a fever and/or respiratory symptoms. Patients diagnosed with COVID-19 during admission were isolated in single-occupancy rooms with negative pressure when available, otherwise, single-occupancy rooms without negative pressure were used. Healthcare workers adhered to standard, contact, and droplet precautions for all COVID-19 patients. Airborne precautions were implemented during aerosol-generating procedures. Personal protective equipment included KF94 or equivalent respirators, face shields or goggles, non-sterile gloves, and isolation gowns. During aerosol-generating procedures, N95 or equivalent respirators were used.

When COVID-19 was confirmed in a patient in a multiple-occupancy room, all patients sharing the room were tested with the SARS-CoV-2 PCR assay during the infectious window (defined as 48 h before symptom onset or a positive test in the absence of symptoms). Exposed roommates were placed on droplet precautions if they were inpatients, or on home quarantine if they were being discharged, for 14 days after their last exposure. Considering the median incubation period < 7 days, the quarantine period was reduced to 7 days during the late study period.

### Definitions

A case of COVID-19 was defined as a positive SARS-CoV-2 PCR assay result using any respiratory specimens. Patients with a recent history of infection were categorized according to national guidelines, which were based on the Centers for Disease Control and Prevention protocol, as follows [20, 21]. Reinfection was defined as a positive test more than 90 days after the last diagnosis (with or without symptoms), a positive test 45–89 days after the last diagnosis (with symptoms), or a history of exposure to a patient with a confirmed positive test result. All other cases were classified as re-positivity. Cases were classified as nosocomial if diagnosed more than 5 days after hospitalization.

Hospital rooms were classified as 1A, 1B, 2, 3, 4, 5, or 6 according to the number of patients per room. 1A refers to a single-bed room in an all single-bed room ward, whereas 1B refers to a single-bed room in a mixed single- and multi-bed room ward.

### Patients

We retrospectively reviewed the hospitalization periods of adult patients aged ≥ 18 years from January 1, 2022, to December 31, 2022. All hospitalization periods were divided according to the hospital room type. Hospitalization periods were excluded from the analysis based on the following criteria.

1. If the length of stay in one hospital room was < 5 days, the hospitalization period for that room was excluded.
2. Hospitalization periods in intensive care units (ICUs) were excluded.
3. Hospitalization periods after the diagnosis of nosocomial COVID-19 (including periods at the time of re-admission) were excluded.
4. If nosocomial COVID-19 was diagnosed within 5 days after a room change, hospitalization periods in the pre-and post-movement rooms were excluded.
5. Hospitalization periods for patients with community-acquired COVID-19 and those with re-positivity results were excluded.

If a patient was hospitalized multiple times during the study period, each hospitalization was included in the analysis.

### Variables

The following variables were extracted from SUPREME^®^, a clinical data warehouse at the study hospital: age, sex, underlying diseases, date of admission, date of discharge, hospitalization room, and SARS-CoV-2 reverse-transcription PCR assay results. Underlying disease data were extracted using International Classification of Diseases 10th revision codes, including diabetes mellitus, chronic kidney disease, cardiovascular disease, heart failure, cerebrovascular accident, liver cirrhosis, chronic obstructive pulmonary disease, interstitial lung disease, rheumatologic disease, asthma, hematologic malignancy, solid malignancy, solid organ transplantation, and hematopoietic stem cell transplantation. Patients were considered vaccinated if they had completed the primary series or received booster vaccinations [22].

### Statistical analysis

Patients’ baseline characteristics were compared across all study groups using the absolute standardized difference (ASD). ASDs of < 0.1 and > 0.25 indicated negligible and large differences, respectively, in the mean or proportion of covariates between two groups [23]. Statistical significance was defined as a mean ASD of > 0.15 and maximum ASD of > 0.3.

To estimate the incidence rates of nosocomial COVID-19 per room type, the hospitalization period per room was used to calculate the follow-up time when estimating the incidence, with the hospitalization period per room regarded as the analysis unit. The incidence rate was defined as the sum of nosocomial COVID-19 incident cases divided by the total follow-up time. A Poisson regression model was used to test the trend in incidence rate of nosocomial COVID-19 according to the number of patients per room.

The association between the number of patients per room and the risk of nosocomial COVID-19 was estimated using a Cox proportional hazards regression model. Age, sex, and underlying diseases were included in the multivariable model.

Although the vaccination status was an important variable, it could not be extracted from the database of the clinical data warehouse, and it was not feasible to check the vaccination histories of all patients. As an alternative, we reviewed the vaccination histories of all patients with confirmed nosocomial COVID-19. Based on these results, we assumed the vaccination rate of the remaining patients and calculated the number of patients required to estimate the vaccination rate using a precision rate of 5% and the 95% confidence interval (CI). We then reviewed the vaccination histories of the remaining randomly sampled patients. The weighted vaccination rates according to room type were estimated via multiplication of the vaccination rates of patients with and without nosocomial COVID-19 by their sampling weights. Sampling weights were calculated as the inverse of the sampling fraction (number of data points with vaccination information/number of analysis data) per room type and nosocomial COVID-19 status.

Subgroup analysis was performed among patients with known vaccination information to determine the association, adjusted for vaccination status and the above-listed variables. The association was estimated by fitting a Cox proportional hazards model, weighted using the sampling weight.

We also performed sensitivity analysis using a diagnostic cut-off for nosocomial COVID-19 set at 10 days after the date of admission.

Statistical analyses were conducted with support from the Medical Research Collaboration Center and performed using SAS version 9.4 (SAS Institute Inc., Cary, NC, USA), IBM SPSS Statistics for Windows, version 28.0 (IBM Corp., Armonk, NY, USA), and PASS 2022, v22.0.2 (NCSS, LLC, Kaysville, UT, USA). The threshold for statistical significance was regarded as *P* < 0.05.

### Ethics

This study protocol was approved by the Institutional Review Board (IRB No. H-2308-016-1454) and Data Review Board (DRB No. DRB-E(I)-2023-08-07) of Seoul National University Hospital. The requirement for informed consent was waived because of the retrospective nature of the study.

## Results

### Study cohort

**Fig. 1:**
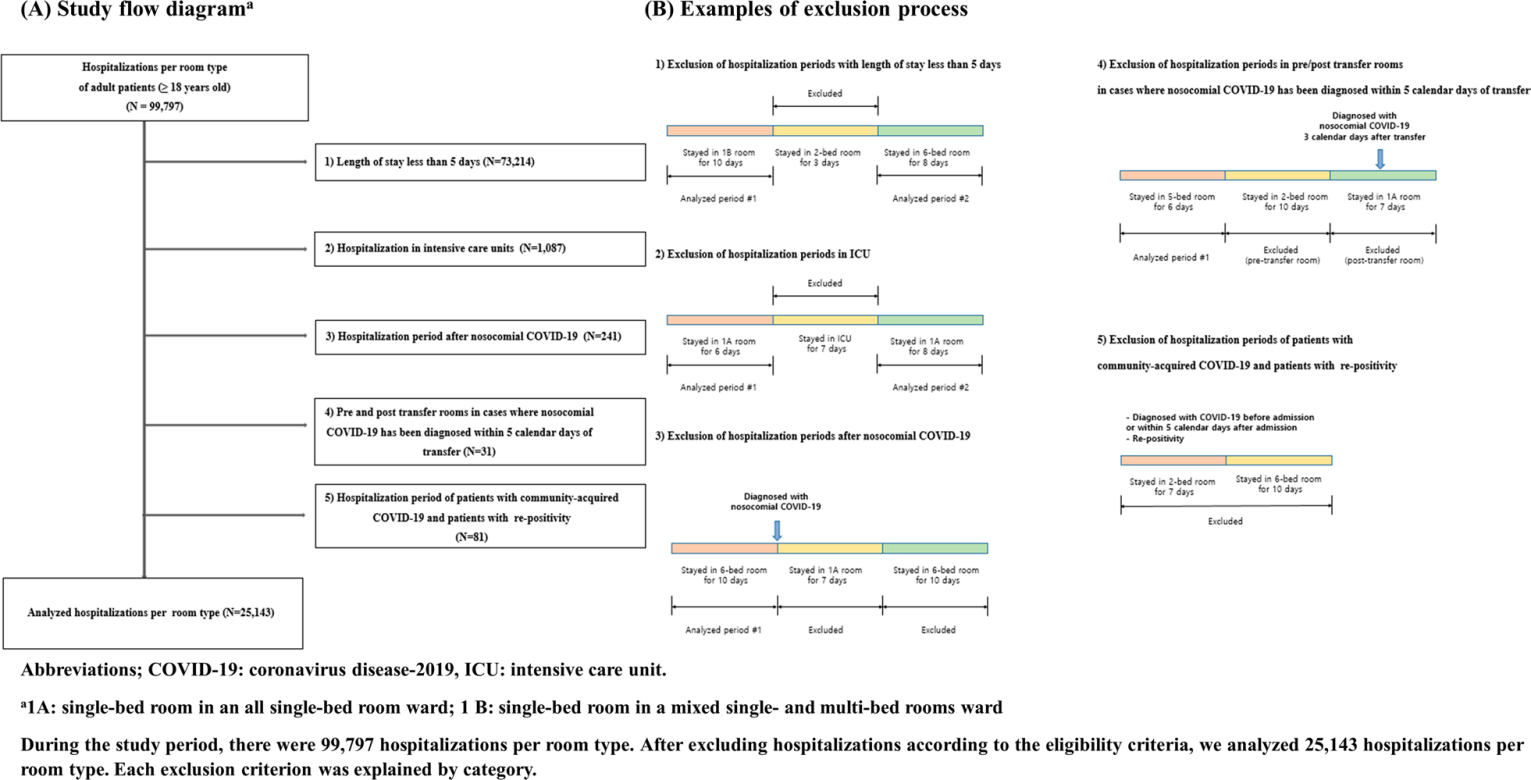
Study flow diagram and examples of exclusion process. Abbreviations; COVID-19: coronavirus disease-2019, ICU: intensive care unit. ^a^1A: single-bed room in an all single-bed room ward; 1B: single-bed room in a mixed single- and multi-bed rooms ward. During the study period, there were 99,797 hospitalizations per room type. After excluding hospitalizations according to the eligibility criteria, we analyzed 25,143 hospitalizations per room type. Each exclusion criterion was explained by category

During the study period, 80,702 patients aged ≥ 18 years were hospitalized. Of these patients, 67,890 stayed in only one room type during hospitalization; 12,812 (15.9%) were transferred and stayed in two or more room types during hospitalization. Considering room transfers, 99,797 hospitalizations were analyzed. Among these hospitalizations, we excluded those for which the length of stay was < 5 days (n = 73,214), admissions to intensive care units (n = 1087), hospitalization periods occurring after nosocomial COVID-19 (n = 241), those for which the hospitalization room was a pre- or post-transfer room when nosocomial COVID-19 had been diagnosed within 5 days of transfer (n = 31), and those in which patients were diagnosed with community-acquired COVID-19 or had re-positivity results (n = 81). Finally, 22,757 hospitalizations of 18,577 patients remained. Among these, 1918 (8.4%) patients underwent room transfers, and 25,143 hospitalizations per room type were analyzed (Fig. 1).

### Demographic, baseline characteristics, and vaccination status

The number of hospitalizations per room type and the patients’ baseline characteristics are shown in Table 1. Seven baseline covariates (age, sex, diabetes mellitus, chronic kidney disease, cardiovascular disease, solid malignancy, and duration of hospitalization) showed large standardized differences regarding means or proportions (mean ASD > 0.15 and maximum ASD > 0.3).

**Table 1:** Patients’ baseline characteristics according to number of patients per room type

Variables^a^	1A (n = 268)	1B (n = 1481)	2 (n = 6743)	3 (n = 606)	4 (n = 3001)	5 (n = 1935)	6 (n = 11,109)	Mean ASD^c^	Maximum ASD^c^
*Hospitalizations* *per* *room* *type*^*b*^ (*total* *n = *25,143)									
Age, years	68 [56–78.5]	62 [49–72]	61 [48–70]	52 [29–65]	63 [51–71]	61 [49–70]	63 [50–72]	0.31	0.95
Sex, male	148 (55.2)	790 (53.3)	3365 (49.9)	289 (47.7)	1075 (35.8)	643 (33.2)	6224 (56.0)	0.22	0.47
Diabetes mellitus	63 (23.5)	310 (20.9)	1033 (15.3)	63 (10.4)	517 (17.2)	225 (11.6)	1876 (16.9)	0.15	0.35
Chronic kidney disease	5 (1.9)	217 (14.7)	359 (5.3)	4 (0.7)	128 (4.3)	28 (1.4)	492 (4.4)	0.22	0.55
Cardiovascular disease	29 (10.8)	179 (12.1)	545 (8.1)	15 (2.5)	248 (8.3)	96 (5.0)	966 (8.7)	0.15	0.38
Heart failure	8 (3.0)	65 (4.4)	236 (3.5)	8 (1.3)	137 (4.6)	40 (2.1)	428 (3.9)	0.09	0.19
Cerebrovascular accident	29 (10.8)	156 (10.5)	408 (6.1)	21 (3.5)	272 (9.1)	128 (6.6)	890 (8.0)	0.12	0.29
Liver cirrhosis	19 (7.1)	69 (4.7)	440 (6.5)	18 (3.0)	223 (7.4)	48 (2.5)	684 (6.2)	0.11	0.23
COPD	10 (3.7)	34 (2.3)	137 (2.0)	11 (1.8)	79 (2.6)	44 (2.3)	298 (2.7)	0.04	0.12
Interstitial lung disease	19 (7.1)	46 (3.1)	140 (2.1)	11 (1.8)	108 (3.6)	82 (4.2)	306 (2.8)	0.10	0.26
Rheumatologic disease	15 (5.6)	57 (3.8)	163 (2.4)	10 (1.7)	118 (3.9)	37 (1.9)	460 (4.1)	0.09	0.21
Asthma	21 (7.8)	52 (3.5)	164 (2.4)	15 (2.5)	101 (3.4)	35 (1.8)	296 (2.7)	0.10	0.28
Hematologic malignancy	20 (7.5)	116 (7.8)	477 (7.1)	35 (5.8)	304 (10.1)	177 (9.1)	727 (6.5)	0.07	0.16
Solid malignancy	165 (61.6)	651 (44.0)	3559 (52.8)	242 (40.0)	1516 (50.5)	1434 (74.1)	4992 (44.9)	0.29	0.74
SOT	8 (3.0)	70 (4.7)	231 (3.4)	2 (0.3)	119 (4.0)	32 (1.7)	378 (3.4)	0.11	0.28
HSCT	0 (0.0)	5 (0.3)	12 (0.2)	0 (0.0)	18 (0.6)	5 (0.3)	39 (0.4)	0.05	0.11
Duration of hospitalization	8 [6–15]	12 [7–23]	9 [6–17]	16.5 [10–28]	9 [6–18]	7 [6–15]	9 [6–15]	0.17	0.41

Data are presented as n (%) or median [interquartile range] unless otherwise indicated

ASD, absolute standardized difference; COPD, chronic obstructive pulmonary disease; SOT, solid organ transplantationl; HSCT, hematopoietic stem cell transplantation

^a^1A: single-bed room in an all single-bed room ward; 1B: single-bed room in a mixed single- and multiple-bed room ward

^b^If a patient was hospitalized in multiple hospital rooms, hospitalization period in each room was counted separately

^c^Mean ASD and maximum ASD are the mean and maximum of the 21 pairwise ASDs among the seven groups, respectively

The vaccination rate among patients with nosocomial COVID-19 ranged from 0.0 to 85.1% (Table 2a). Based on this finding, we assumed a vaccination rate of 80% for the remaining patients and calculated that 246 patients per room type would be required to estimate the vaccination rate with a precision rate of 5% and the 95% CI. Among the randomly sampled 246 patients without nosocomial COVID-19, the vaccination rate ranged from 78.0 to 89.8% (Table 2b). The estimated vaccination rates per room type were as follows: 1A rooms, 88.8% (95% CI 84.7–92.8); 1B rooms, 84.2% (95% CI 79.7–88.7); 2-bed rooms, 88.0% (95% CI 84.0–91.9); 3-bed rooms, 89.3% (95% CI 85.6–93.1); 4-bed rooms, 77.7% (95% CI 72.6–82.8); 5-bed rooms, 90.3% (95% CI 86.7–93.9); and 6-bed rooms, 88.1% (95% CI 84.2–92.1) (Table 2c). Overall, vaccination coverage did not significantly differ between patients in single- and multiple-occupancy rooms; however, patients in 4-bed rooms had a lower vaccination rate than patients in the other rooms (*P* < 0.001).

**Table 2:** Vaccination status according to number of patients per room

Number of patients per room^a^	Number	Unvaccinated	Vaccinated	
			Partially vaccinated	Completed primary series or boosted
*(a)* *Vaccination* *status* *of* *patients* *with* *nosocomial* *COVID-19*				
1A	1	1 (100.0)	0 (0.0)	0 (0.0)
1B	13	1 (7.7)	1 (7.7)	11 (84.6)
2	72	21 (29.2)	0 (0.0)	51 (70.8)
3	9	3 (33.3)	0 (0.0)	6 (66.7)
4	40	6 (15.0)	1 (2.5)	33 (82.5)
5	31	8 (25.8)	0 (0.0)	23 (74.2)
6	235	30 (12.8)	5 (2.1)	200 (85.1)
Total	401	70 (17.5)	7 (1.7)	324 (80.8)

Number of patients per room^a^	Number	Extraction rate	Unvaccinated	Vaccinated	
				Partially vaccinated	Completed primary series or boosted
*(b)* *Vaccination* *status* *of* *randomly* *sampled* *patients* *without* *nosocomial* *COVID-19*					
1A	246	246/266	17 (6.9)	9 (3.7)	220 (89.4)
1B	246	246/1456	36 (14.6)	3 (1.2)	207 (84.1)
2	246	246/6646	28 (11.4)	2 (0.8)	216 (87.8)
3	246	246/593	22 (8.9)	3 (1.2)	221 (89.8)
4	246	246/2941	41 (16.7)	13 (5.3)	192 (78.0)
5	246	246/1893	24 (9.8)	1 (0.4)	221 (89.8)
6	246	246/10,840	24 (9.8)	4 (1.6)	218 (88.6)
Total	1722	1722/24,635	192 (11.1)	35 (2.0)	1495 (86.8)

Number of patients per room^a^	Patients with nosocomial COVID-19		Patients without nosocomial COVID-19		Estimated vaccination rate^b^ (95% CI)
	Number	Vaccinated (%)	Number	Vaccinated (%)	
*(c)* *Estimation* *of* *vaccination* *rates*					
1A	1	0.0	246	89.4	88.8 (84.7–92.8)
1B	13	84.6	246	84.1	84.2 (79.7–88.7)
2	72	70.8	246	88.2	88.0 (84.0–91.9)
3	9	66.7	246	89.8	89.3 (85.6–93.1)
4	40	82.5	246	77.6	77.7 (72.6–82.8)
5	31	74.2	246	90.7	90.3 (86.7–93.9)
6	235	85.1	246	88.2	88.1 (84.2–92.1)
Total	401	80.8	1722	86.9	86.8 (84.6–89.0)

Data are presented as n (%) unless otherwise indicated

COVID-19, coronavirus disease 2019; CI, confidence interval

^a^1A: single-bed room in an all single-bed room ward; 1B: single-bed room in a mixed single- and multiple-bed room ward

^b^Vaccination rates were estimated using the weighted vaccination rate: the vaccination rates of patients with and without nosocomial COVID-19 were multiplied by their sampling weights

### Nosocomial COVID-19

During the 138,997 patient-days of observation, 401 cases of nosocomial COVID-19 were diagnosed. The incidence rate of nosocomial COVID-19 tended to increase according to the number of patients per room, ranging from 3.05 to 38.64 cases per 10,000 patient-days in single- to 6-bed rooms, respectively (*P* < 0.001, Table 3).

**Table 3:** Incidence rate of nosocomial COVID-19 according to number of patients per room

Number of patients per room^a^	Number of hospitalizations	Number of patients with nosocomial COVID-19	Patient-days of observation	Incidence rate per 10,000 patient-days
1A	268	1	3283	3.05
1B	1481	13	8892	14.62
2	6743	72	35,825	20.10
3	606	9	5469	16.46
4	3001	40	16,480	24.27
5	1935	31	8238	37.63
6	11,109	235	60,810	38.64
Total	25,143	401	138,997	28.85

COVID-19, coronavirus disease 2019

^a^1A: single-bed room in an all single-bed room ward; 1B: single-bed room in a mixed single- and multiple-bed room ward

### Risk of nosocomial COVID-19 based on the number of patients per room

The results of multivariable Cox proportional hazards regression are shown in Table 4. Using 1B rooms as the reference, we observed an increasing trend in the hazard ratios of nosocomial COVID-19 according to the number of patients per room from 0.14 for 1A rooms to 2.66 for 6-bed rooms (*P* < 0.001). Furthermore, the hazard ratios were significantly higher for rooms with ≥ 5 patients than for 1B rooms.

**Table 4:** Results of Cox proportional hazards regression model for association between number of patients per room and nosocomial COVID-19

Number of patients per room^a^	Univariable analysis		Multivariable analysis	
	HR (95% CI)	*P*-value	HR (95% CI)	*P*-value
1A	0.15 (0.001–1.10)	0.184	0.14 (0.001–1.03)	0.168
1B	1.00 (Ref)		1.00 (Ref)	
2	1.30 (0.75–2.44)	0.373	1.41 (0.82–2.65)	0.247
3	1.18 (0.50–2.68)	0.698	1.39 (0.58–3.20)	0.447
4	1.60 (0.89–3.07)	0.137	1.74 (0.96–3.35)	0.080
5	2.37 (1.28–4.64)	0.008	2.68 (1.44–5.28)	0.003
6	2.53 (1.52–4.60)	0.001	2.66 (1.60–4.85)	< 0.001

COVID-19, coronavirus disease 2019; HR, hazard ratio; CI, confidence interval

^a^1A: single-bed room in an all single-bed room ward; 1B: single-bed room in a mixed single- and multiple-bed room ward

Subgroup analysis, focusing solely on 2627 patients with a known vaccination status, also revealed an increasing trend in the hazard ratio of nosocomial COVID-19 according to the number of patients per room (Supplementary Table 1).

The results of sensitivity analysis, using a diagnostic cut-off for nosocomial COVID-19 set at 10 days after the date of admission, are shown in Supplementary Table 2. The tendency for the risk of nosocomial COVID-19 to increase according to the number of patients per room persisted regardless of the definition of nosocomial COVID-19.

## Discussion

Higher nosocomial COVID-19 rates were detected among patients in multiple-occupancy rooms than among those in single-occupancy rooms. A dose–response relationship was present between the number of patients in a room and the incidence of nosocomial COVID-19. These findings suggest a strong correlation between treatments in multiple-occupancy rooms and the acquisition of SARS-CoV-2 infection.

This study was conducted in Korea in 2022. The prevalence of COVID-19 was relatively low in Korea until late 2021 because of aggressive testing, contact tracing, strict quarantine policies, and high vaccination rates. Despite the high vaccination rates, the prevalence abruptly increased in February 2022 due to the emergence of highly transmissible Omicron variants [24, 25]. The incidence of nosocomial COVID-19 increased during the community-wide Omicron outbreak compared with the Delta outbreak [26, 27]. We believe that the predominance of highly transmissible Omicron variants in the community highlights the impact of multiple-occupancy rooms on nosocomial COVID-19.

We applied several exclusion criteria, some of which require explanation. ICU stays were excluded due to distinct differences in patient care compared to general wards. The ICU was an open shared space with 10–25 beds, lower patient-to-nurse ratio, and higher patient turnover compared to general wards. In addition, hospitalization periods in pre-and post-movement rooms were excluded when nosocomial COVID-19 was diagnosed within 5 days of a room change. Considering the SARS CoV-2 incubation period of 2–14 days, it was unclear whether transmission occurred before or after the room change. To minimize misclassification, the pre-movement period was excluded.

The criteria for defining nosocomial COVID-19 have not yet been standardized. The incubation period of wild type SARS-CoV-2 ranges from 2 to 14 days (median, 5.1 days) [28], and that of the Omicron variant is shorter [29, 30]. In this study, we selected 5 days after hospitalization as the cut-off for diagnosing nosocomial COVID-19 to cover the median incubation period for COVID-19; this approach also avoided underestimating the incidence of nosocomial COVID-19 [28]. Other studies also defined nosocomial COVID-19 as a positive SARS-CoV-2 PCR result 5 days after admission in patients who had a negative PCR result on admission [14, 31]. When we separately analyzed the data using 10 days as the cut-off (which encompassed 95% of the incubation period), the trends were consistent (Supplementary Table 2).

SARS-CoV-2 mainly spreads through respiratory droplets and/or aerosols; it less frequently spreads through environmental contamination [32, 33]. The spread of SARS-CoV-2 after exposure to rooms with multiple occupancies has also been reported [1, 2, 15, 34–36]. The rate of a second attack rate after exposure to SARS-CoV-2 in a shared room ranges from 19 to 40% [15, 16, 34]. Interventions performed to interrupt the nosocomial spread of respiratory viruses include rapid detection and isolation of patients with transmissible viruses, proper hand hygiene, improved ventilation, implementation of universal masking, and vaccination policies for patients and healthcare personnel [3, 4]. Efforts to minimize the use of multiple-occupancy rooms are needed to reduce the nosocomial spread of pathogens transmitted by respiratory secretions. In a prospective observational study, double- or multi-occupancy rooms were independently associated with nosocomial influenza compared with single-occupancy rooms (adjusted odds ratio 3.42; 95% confidence interval 1.29–9.08) [37]. Another study showed that the relative risk of nosocomial influenza was 2.67 (95% confidence interval 1.05–6.76) in double-occupancy rooms compared with single-occupancy rooms [38]. We found that the incidence of nosocomial COVID-19 increased according to the number of patients in a room. Patients in shared rooms have minimal close contact with their roommates. Therefore, transmission to roommates might occur via respiratory droplets or aerosols despite universal masking of patients, curtains between patients, and a mean separation distance of 7 feet. A higher number of patients in a room is associated with greater risk of exposure to patients with asymptomatic or symptomatic COVID-19. The incidence of nosocomial COVID-19 was lowest in wards containing only single-bed rooms (1A ward). This suggests that less crowded wards are beneficial for reducing the spread of nosocomial COVID-19. If a patient in a multi-occupancy room had fever or respiratory symptoms in the present study, diagnostic tests were immediately performed to detect COVID-19 and isolate patients with newly detected COVID-19. To minimize nosocomial transmission, droplet precautions were implemented for roommates of COVID-19 patients for 14 days, consistent with the longest incubation period of SARS-CoV-2. However, such efforts are insufficient to prevent the transmission of SARS-CoV-2 in multiple-occupancy rooms because nearly 60% of SARS-CoV-2 transmissions are attributable to asymptomatic or pre-symptomatic individuals [39]. Several published guidelines recommend single-occupancy rooms for refurbished or new hospital wards [40, 41]. The proportion of single-occupancy hospital rooms has increased in many countries [42, 43]. We suggest that an increased proportion of single-occupancy rooms is necessary to reduce the spread of nosocomial infections caused by respiratory droplets and/or aerosols.

Although this study demonstrated the impact of multiple-occupancy rooms on the nosocomial spread of COVID-19, it had several limitations. First, we did not analyze genetic relationships of SARS-CoV-2 via molecular methods to confirm spread in shared rooms. Some patients may have been infected by people other than their roommates. Second, we could not investigate the vaccination histories of all patients, although the vaccination rate is an important factor influencing the incidence of nosocomial COVID-19. To minimize this limitation, we examined the vaccination histories of all patients with confirmed COVID-19; we found no significant differences between patients in single- or multiple-occupancy rooms. We also performed a separate analysis of 2627 patients whose vaccination history information was available; the results were consistent with the initial analysis. Third, as mentioned above, the cut-off days to define nosocomial COVID-19 were not standardized. To minimize this limitation, we analyzed data using 10 days as the cut-off; the results were consistent with the initial analysis. Fourth, patients diagnosed with nosocomial COVID-19 after discharge may have been excluded. Fifth, as shown in Fig. 1, a significant number of hospitalization periods were excluded to minimize misclassification. Although this reduced the sample size, the focus on patients with confidently determined nosocomial spread was prioritized. Considering the year-long study duration, a sufficient number of patients and observation time remained.

## Conclusion

We have demonstrated that multiple-occupancy rooms play a role in the spread of nosocomial COVID-19. We suggest minimizing the use of multiple-occupancy rooms to facilitate infection control, especially concerning the spread of respiratory viruses within hospitals.

### Supplementary Information

Additional file 1.

## Data Availability

The data that support the findings of this study are available upon reasonable request.
