# Supplementary material for: Risk of nosocomial coronavirus disease 2019: comparison between single- and multiple-occupancy rooms
Source: Antimicrob Resist Infect Control. 2024 Aug 30;13:95. doi: 10.1186/s13756-024-01454-w (PMC11365205; doi:10.1186/s13756-024-01454-w)
Supplement: Supplementary file 1 — Additional file 1. [file 13756_2024_1454_MOESM1_ESM.docx]

**Supplementary Table 1.**

**(a) Incidence rate of nosocomial COVID-19 according to number of patients per room only among patients with known vaccination history (n = 2,627)**

| **Number of patients per room^a^** | **Number of hospitalizations** | **Number of patients with nosocomial COVID-19** | **Patient-days of observation** | **Incidence rate^b^**  **per 10,000 patient-days** |
| --- | --- | --- | --- | --- |
| 1A | 254 | 1 | 2977 | 3.15 |
| 1B | 345 | 13 | 2760 | 10.91 |
| 2 | 462 | 72 | 3297 | 16.70 |
| 3 | 337 | 9 | 3143 | 14.34 |
| 4 | 348 | 40 | 2120 | 23.69 |
| 5 | 311 | 31 | 1406 | 37.93 |
| 6 | 570 | 235 | 4172 | 38.22 |
| Total | 2627 | 401 | 19,875 | 26.57 |

COVID-19: coronavirus disease 2019

^a^1A: single-bed room in an all single-bed room ward; 1B: single-bed room in a mixed single- and multiple-bed room ward

^b^Incidence rates were calculated by applying sampling weights to cases with vaccination information to adjust for unequal sample selection probabilities.

**(b) Results of Cox proportional hazards regression for association between number of patients per room and nosocomial COVID-19 only among patients with known vaccination history (n = 2,627)**

| **Number of patients per room^a^** | **Multivariable analysis** | |
| --- | --- | --- |
|  | **HR (95% CI)** | **P-value** |
| 1A | 0.17 (0.01–2.88) | 0.218 |
| 1B | 1.00 (Ref) |  |
| 2 | 1.43 (0.79–2.62) | 0.240 |
| 3 | 1.27 (0.54–2.98) | 0.591 |
| 4 | 2.20 (1.17–4.16) | 0.015 |
| 5 | 3.23 (1.65–6.30) | 0.001 |
| 6 | 3.44 (1.95–6.07) | <0.001 |

COVID-19: coronavirus disease 2019, HR: hazard ratio, CI: confidence interval

^a^1A: single-bed room in an all single-bed room ward; 1B: single-bed room in a mixed single- and multiple-bed room ward

**Supplementary Table 2.**

**(a) Incidence rate of nosocomial COVID-19 according to number of patients per room** **with diagnostic cut-off for nosocomial COVID-19 set to 10 calendar days after date of admission**

| **Number of patients per room^a^** | **Number of hospitalizations** | **Number of patients with nosocomial COVID-19** | **Patient-days of observation** | **Incidence rate**  **per 10,000 patient-days** |
| --- | --- | --- | --- | --- |
| 1A | 100 | 1 | 3053 | 3.28 |
| 1B | 886 | 12 | 8198 | 14.64 |
| 2 | 3252 | 62 | 31,263 | 19.83 |
| 3 | 455 | 8 | 5211 | 15.35 |
| 4 | 1419 | 35 | 14,501 | 24.14 |
| 5 | 717 | 27 | 6892 | 39.18 |
| 6 | 4737 | 194 | 51,318 | 37.80 |
| Total | 11,566 | 339 | 120,436 | 28.15 |

COVID-19: coronavirus disease 2019

^a^1A: single-bed room in an all single-bed room ward; 1B: single-bed room in a mixed single- and multiple-bed room ward

**(b) Results of Cox proportional hazards regression for association between number of patients per room and nosocomial COVID-19 with diagnostic cut-off for nosocomial COVID-19 set to 10 calendar days after date of admission**

| **Number of patients per room^a^** | **Multivariable analysis** | |
| --- | --- | --- |
|  | **HR (95% CI)** | **P-value** |
| 1A | 0.15 (0.00–1.16) | 0.193 |
| 1B | 1.00 (Ref) |  |
| 2 | 1.42 (0.80–2.73) | 0.266 |
| 3 | 1.32 (0.52–3.16) | 0.543 |
| 4 | 1.79 (0.96–3.55) | 0.080 |
| 5 | 2.90 (1.51–5.91) | 0.002 |
| 6 | 2.64 (1.55–4.94) | 0.001 |

^a^1A: single-bed room in an all single-bed room ward; 1B: single-bed room in a mixed single- and multiple-bed room ward

COVID-19: coronavirus disease 2019, HR: hazard ratio, CI: confidence interval
